# Between-Season Changes in the Cycling Power Profile in Relation to Training Volume and Moderate-to-High Intensity in International Junior and U23 Triathletes: A Longitudinal Study

**DOI:** 10.3390/jfmk11020138

**Published:** 2026-03-26

**Authors:** Raúl Espejo, Jesús Martínez-Sobrino, Jesús Santos del Cerro, Santiago Veiga

**Affiliations:** 1 Departamento de Deportes, Facultad de Ciencias de la Actividad Física y del Deporte (INEF), Universidad Politécnica de Madrid, 28040 Madrid, Spain; raul_espejo94@hotmail.es (R.E.); santiago.veiga@upm.es (S.V.); 2 Department of Statistics, University of Castilla-La Mancha, 45004 Toledo, Spain; jesus.scerro@uclm.es

**Keywords:** training load, performance, monitoring, endurance

## Abstract

**Background:** The power profile is a reliable tool for monitoring performance in the cycling segment of triathlon. This study aimed to analyze the evolution of Mean Maximal Power (MMP) in international triathletes and to examine its relationship with external load-based training characteristics. **Methods**: Cycling training and competition data from 14 junior and U23 international triathletes (seven males: 21 ± 1 years, 69 ± 3 kg, and 181 ± 7 cm; seven females: 22 ± 3 years, 54 ± 5 kg, and 166 ± 3 cm) were analyzed longitudinally for three consecutive seasons. The MMP from the power profile was recorded, along with the training volume accumulated in each 2.0 W·kg^−1^ power band. **Results**: All the MMP values, except values of 10 s, 30 s and 5 min, increased (*p* < 0.05) over the three seasons (Δ = 0.9% to 4.8%; ES = 0.30–0.47), as did the total time (Δ = 22.1%; ES = 0.42) and total distance (Δ = 32.8%; ES = 0.61). Specifically, the percentage of time spent in the 4–6 W·kg^−1^ power band (ES = 0.42) and MMP values for 1–20 min durations (ES = 0.25–0.47) increased (*p* < 0.05) from the second to the third season. MMP values ≤ 30 s showed a very large correlation (above r = 0.74) with the percentage of time spent in power bands of 12–14 W·kg^−1^. All the MMP values showed a negative correlation with the percentage of time spent in the 0–2 W·kg^−1^ power band. **Conclusions**: Improvements in MMP ≥ 1 min values over consecutive seasons were associated with greater total training volume and time spent in moderate-intensity power bands, whereas MMP ≤ 30 s were linked to very high-intensity power outputs.

## 1. Introduction

Triathlon, an endurance sport consisting of three sequential disciplines—swimming, cycling, and running—has grown in popularity since its debut in the Sydney 2000 Olympic Games. Its race tactics are particularly relevant in the cycling segment, where external environmental factors such as wind [1], the technical difficulty of the course [2,3] and drafting conditions [4] influence the demands of competition.

Scientific research related to the professional practice of cycling has advanced in recent years with the use of power meters that are extensively utilized both in triathlon [4,5,6] and also road cycling [7,8]. The power profile, represented by the Mean Maximal Power (MMP) over a given time period, has proven to be a valuable tool for analyzing and monitoring a cyclist’s peak performance [9] during training sessions, competitions or competitive seasons [10]. Recent studies have observed that a 3% improvement in the MMP between two different seasons would be within the expected ranges for professional cyclists [11].

In line with other sport disciplines, the assessment of training load in cycling through the quantification of the volume and intensity of the different training zones is important for analyzing the athlete’s adaptations and for relating the training variables to performance [12]. The periodization of the training load components—defined as volume, intensity, and frequency—through the season in endurance sports can be structured according to different-intensity distribution models. A polarized approach prioritizes alternating high-intensity sessions (zone 3 or above the second ventilatory threshold) with low-intensity work (zone 1 or below the first ventilatory threshold), generally combined with a high training volume [13,14]. In contrast, a pyramidal model places greater emphasis on moderate-intensity training (zone 2 or between ventilatory thresholds), especially during specific stages of the Olympic cycle [15]. In support of this approach, recent evidence indicates that elite cyclists typically follow a pyramid-shaped training distribution across the season—with greater emphasis on high-intensity sessions during competition periods—as it allows for a more balanced management of training volume and intensity [16,17,18]. To date, there is no consensus on which intensity distribution is the most effective for performance in endurance sports, but the different types of adaptations observed for the distinct training intensities are acknowledged [19]. A recent study on trained cyclists observed improvements in their lactate threshold at 4.0 mmol and maximal oxygen consumption power with a moderate-intensity training (MIT) block, achieving similar improvements than previously reported for high-intensity training (HIT) blocks [20].

The relationship between external training load parameters and power profiles in cycling is quite unknown. Leo et al. (2020) [21] reported in relation to a professional U23 cyclist that increasing the work and training time below the first ventilatory threshold and above the second ventilatory threshold correlated with an improvement in the MMP of 2, 5 and 12 min over the course of a season. However, there is no further evidence on how MMP values could be enhanced through the manipulation of training load, despite reports of variations in training volume in cycling over the course of a season (Δ = 31.5%) and between seasons in triathlon (Δ = 14.8%) [21,22]. Therefore, this study aimed to analyze the evolution of the MMP in competitive triathletes between seasons, and to examine its relationship with training characteristics based on the external load. It was hypothesized that MMP improvements would be positively related to an increased in high-intensity training volume.

## 2. Materials and Methods

### 2.1. Participants

In the present study, data were collected from fourteen international junior and U23 triathletes (seven males and seven females). Their mean ± SD age, body mass and height were 21 ± 1 years, 69 ± 3 kg, and 181 ± 7 cm for males, and 22 ± 3 years, 54 ± 5 kg, and 166 ± 3 cm for females. The triathletes were regular competitors in Continental and World Cups, had an average of 5 ± 2 years of international competition experience and gave written informed consent for their race data to be used for research purposes. The inclusion criteria for participants were as follows: (i) at least three years of experience in international competitions; (ii) at least three years without prolonged interruptions in training and competitions. Due to the limited availability of athletes competing at this level, all eligible international junior and U23 triathletes were included in the study. The study was approved by the local University Ethics Committee and adhered to the Declaration of Helsinki (approval code: ADLDDCET00-SVF-HUMANOS-20240207; approval date: 12 February 2024).

### 2.2. Design and Procedures

Power output data during cycling were analyzed for three seasons per triathlete, with each of the seasons beginning 1 November and ending 31 October. The power output data from each season were measured using Assioma Duo power meters from Favero Electronics Srl (Arcade, TV, Italy), which were zero-offset before each use according to the manufacturer’s instructions. The data were uploaded to TrainingPeaks and stored in WKO5 Build 587 software (Boulder, CO, USA) and then were individually inspected in this software to search for and eliminate anomalous data. Anomalous values were defined as non-progressive increases in relative power near the maximum for each duration of effort, likely reflecting measurement or recording errors, and were manually deleted.

The MMP values (W·kg^−1^) for each participant were recorded over different effort durations (10 s, 30 s, 1 min, 5 min, 10 min, 20 min, 40 min, and 1 h), reflecting the competitive demands characterized by predominant bursts of steady effort [5]. Training volume and intensity data were collected in the form of the total time (h), total distance (km), and the proportion of time spent within each 2.0 W·kg^−1^ power output band relative to the total training time across the triathlete’s season. The power bands were defined as follows: low (≤2 W·kg^−1^), moderate (2–6 W·kg^−1^) and high (≥6 W·kg^−1^) intensity.

### 2.3. Statistical Analysis

The data are presented as the mean (±SD). Normality was assessed using the Kolmogorov–Smirnov test for seasonal analysis. A repeated-measure analysis of variance (ANOVA) was conducted, considering the season number (first to third) and the different durations for MMP determination (from 10 s to 1 h) as within-subject factors. For significant effects, the Bonferroni post hoc procedure was used for pairwise comparisons. The MMP values were correlated with the contribution of the total time in each power band using Pearson correlation coefficients. Threshold values of 0.1, 0.3, 0.5, 0.7 and 0.9 represented small, moderate, large, very large and near-perfect correlations [23]. The statistical analysis of the data was performed using IBM SPSS Statistics for Windows, version 21.0 (IBM Corp., Armonk, NY, USA). The significance level for all analyses was set at *p* < 0.05.

## 3. Results

**Figure 1:**
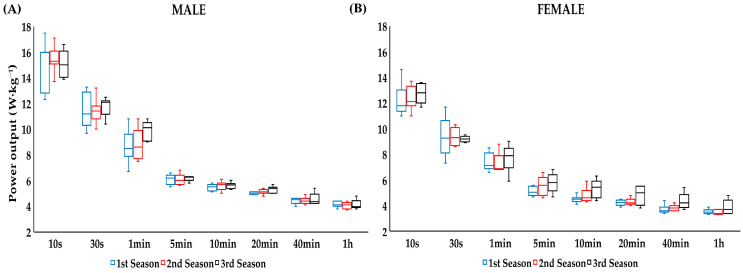
MMP (W·kg^−1^) of seven male (**A**) and female (**B**) international junior and U23 triathletes across three consecutive seasons.

**Figure 2:**
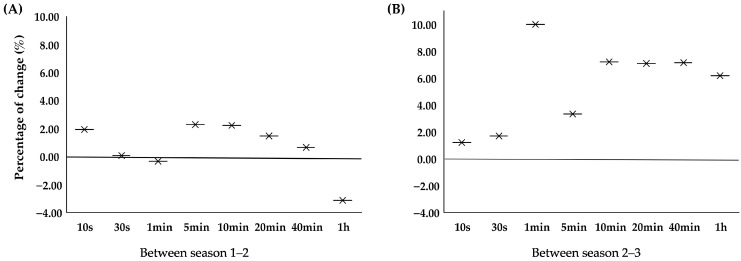
Percentage change in the MMP (W·kg^−1^) between seasons 1 and 2 (**A**) and seasons 2 and 3 (**B**) from fourteen international junior and U23 triathletes.

The MMP recorded for triathletes across seasons was in the range of 15.08 ± 1.04 W·kg^−1^ for males and 12.75 ± 0.75 W·kg^−1^ for females over 10 s, and 4.16 ± 0.22 W·kg^−1^ for males and 3.88 ± 0.59 W·kg^−1^ for females over 1 h (Figure 1A,B) (Table S1). There was a season effect for MMP in all durations (*p* < 0.05; ES = 0.30–0.47), except MMP 10 s, 30 s and 5 min (*p* > 0.05). The percentage change in performance across all the seasons was between 0.9% and 4.8% for durations that were up to one hour of effort. There were no significant differences in any of the MMP from the first to second season (Figure 2A), with the percentage of change in performance ranging from −3.1% to 2.2%, but there were differences from the second to the third season in the MMP for durations of 1 to 20 min (*p* < 0.05), with the percentage change in performance ranging from 3.3% to 10.0% (Figure 2B).

**Figure 3:**
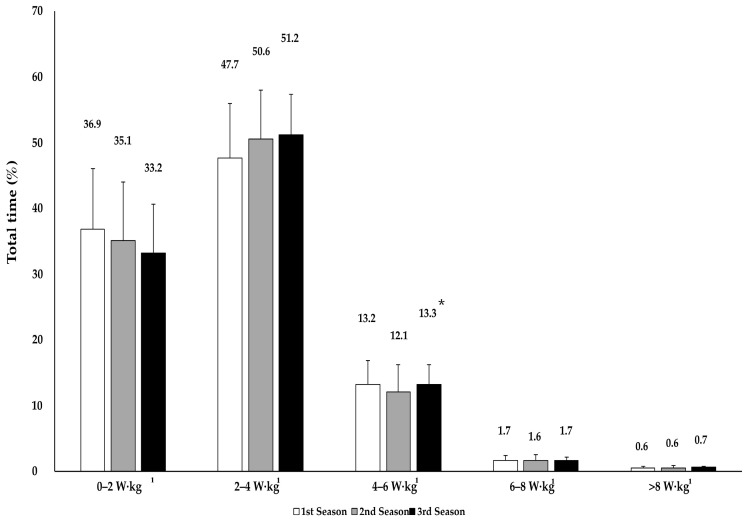
Percentage of time spent by international junior and U23 triathletes during cycling in different power ranges (W·kg^−1^) during three consecutive seasons. * Second season–third season (*p* < 0.05).

Regarding the training variables, the training volume increased from season to season, both in terms of the total time (247 ± 51 h, 269 ± 29 h, and 317 ± 46 h; *p* = 0.03 and ES = 0.42) and total distance (5426 ± 978 km, 6363 ± 1107 km, and 8070 ± 1867 km; *p* = 0.01 and ES = 0.61). Triathletes spent the greatest percentage of time in the 2–4 W·kg^−1^ power band (47.7 ± 5.8%, 50.6 ± 6.8% and 51.2 ± 5.9% for the first, second and third seasons, respectively). There was a season effect in the 4–6 W·kg^−1^ power band (*p* = 0.03; ES = 0.42), with a greater percentage of time spent in the third than in second season (Figure 3).

The MMP values of duration ≤ 30 s showed a very large relationship with the percentage of time spent in high-intensity power bands, particularly 12–14 W·kg^−1^ (r = 0.77 and r = 0.74 for MMP 10 and 30 s, respectively). The MMP value of 1 min showed a large correlation with the time spent in moderate-to-high intensity power bands (≥4 W·kg^−1^) (Table 1). On the other hand, it was observed that MMP values ≥ 5 min had a large correlation with the percentage of time spent in the moderate-intensity (4–6 W·kg^−1^) power band (r = 0.59 to r = 0.64). All the MMP values showed a moderate and large negative correlation with the percentage of time spent in the low-intensity power band (0–2 W·kg^−1^).

In the seasonal analysis, large, very large, and/or nearly perfect correlations were observed between the percentage of time triathletes spent in moderate-to-high intensity power bands (≥4 W·kg^−1^) and all the MMP values, except in the third season for MMP durations ≥ 5 min, which did not show a significant correlation (Table 2). A very large correlation was also observed between the percentage of time in nearly all high-intensity power bands (≥10 W·kg^−1^) and the MMP values for durations of 10 and 30 s across three consecutive seasons (from r = 0.59 to r = 0.92). All the MMP values showed a negative correlation with the percentage of time spent in the low-intensity power band (0–2 W·kg^−1^) in the three consecutive seasons (Table 2).

## 4. Discussion

This study examined the changes in cycling performance (measured as MMP) among international junior and U23 triathletes across consecutive seasons, relating these changes to training load. Our data provide the first longitudinal analysis of the MMP in cycling performance for triathletes across different effort durations and several consecutive seasons, and showed annual improvements of between 0.9% and 4.8% for efforts lasting up to one hour. A greater percentage of time spent in the moderate-intensity power band (4–6 W·kg^−1^) and total training volume related to an improved cycling MMP between seasons.

Our results were higher than the MMP values reported for male U23 cyclists at an MMP of 30 s, 1 min and 10 min compared to our triathletes, while in females a very similar MMP was observed for all durations, with differences ranging between 0.1 and 0.3 W·kg^−1^ for efforts up to 40 min [24]. That may be attributed to the differences between a more controlled laboratory setting and the field tests [25]. The changes in the MMP of young triathletes between seasons revealed smaller changes at short durations than those reported by Valenzuela et al. (2023) [11] in professional cyclists (i.e., 1.5% vs. 5.5% for MMP of 10 s) but similar changes at longer durations (between 2.8% and 3.9%) [26].

For the training external load values, a pyramidal distribution was evident throughout the seasons, characterized by a progressive decrease in relative time, from moderate–low power bands (2–4 W·kg^−1^) to higher-intensity bands (>8 W·kg^−1^). This would support the applicability of a pyramidal model of load distribution in endurance sports [16,18]. The cycling training volume was lower than that recently reported by a nine-time world champion triathlete (155 km vs. 400 km average weekly) [27], which is likely explained by the different stages of sporting development in which triathletes find themselves. On the other hand, the training volume was half (738 h vs. 317 h) of that reported by U23 professional cyclists, along with a lower proportion of training time performed at high intensities—1.9–2.8% in power bands exceeding 6 W·kg^−1^ compared to approximately 8–9% above the second ventilatory threshold [21]. These differences could be explained by the distribution of the training load in triathlon training, where the training volume is distributed among the three disciplines.

The changes in the MMP values in relation to the external training variables showed that triathletes in the present study improved the MMP at 1 to 20 min values from the second to the third season when they both increased their total training volume and the percentage of time in the moderate intensity zone (4–6 W·kg^−1^). These power bands could be associated with relative values around the second ventilatory threshold—located between 4.0 and 5.5 W·kg^−1^ in male triathletes, according to the study by Cejuela & Selles-Perez (2023) [22]—and are consistent with the improvements observed in the lactate threshold at 4.0 mmol and the power at maximal oxygen consumption after an MIT block in trained cyclists [20]. Research conducted with cross-country skiers observed that improvements in aerobic power from junior to world-class level were mainly achieved, in addition to increasing low-intensity training volume, by increasing moderate-intensity training [28]. These findings suggest that moderate-intensity training induces performance improvement in those durations where the aerobic component is more predominant, as in the case of our study.

When examining seasonal changes, the percentage of time spent in moderate and high power bands (≥4 W·kg^−1^) showed a moderate to very large correlation with better MMP values across all analyzed durations (Table 2). Furthermore, MMP values at 1, 10 and 20 min showed the greatest changes in power profile performance (between 2.4% and 3.4%) as the percentage of time in these high-intensity power bands increased. It should be noted that, in our study, power bands above 4 W·kg^−1^ could be located above the second ventilatory threshold, as reported in Cejuela & Selles-Perez’s (2023) [22] study of elite male triathletes. This is in line with the study by Leo et al. (2020) [21], who observed in U23 professional cyclists an improved performance at durations 2, 5 and 12 min of the power profile when increasing training time above the second ventilatory threshold.

Finally, the results shown in our study suggest that increasing the percentage of time spent in power bands ≥ 12 W·kg^−1^, usually identified with anaerobic efforts in triathlon [5], may be related to improvements in the shorter durations (MMP of 10 and 30 s). The physiological adaptations derived from high-intensity efforts, such as sprint interval training (SIT), which activate signaling pathways associated with mitochondrial biogenesis and improve metabolic efficiency, could favor performance in short-duration explosive efforts [19]. In contrast, our analysis showed that performing a greater percentage of time on very low power bands (0–2 W·kg^−1^), both between and within seasons, may not improve the MMP values. In fact, low-intensity training time seems to be identified with periods of rest or non-pedaling, likely resulting from descents or drafting within the group [29]. Consequently, accumulating large training volumes at these intensities may not constitute an effective training stimulus and could lead to detraining, as suggested by the negative correlation observed in our results.

Coaches and triathletes should be aware of variations in cycling performance over several seasons, and its relationship with training load variables. Season-to-season improvements between 0.9% and 4.8% in MMP durations up to 1 h could be expected in international junior and U23 triathletes. This could be associated with an increase of approximately 35 h in total training time, along with a 12–13% contribution in time in the 4–6 W·kg^−1^ power band from one season to the next, in addition to an increase in high-intensity SIT tasks (≥12 W·kg^−1^), which may be associated with improvements in shorter-duration MMP (10 and 30 s). Training adaptations in cycling could be expected above the 2 W·kg^−1^ threshold, since values below could be associated with non-pedaling moments or intensities too low to generate significant stimuli. However, these results could be supported with future intervention studies examining the role of the training intensity on the MMP profile. Some limitations should be acknowledged when interpreting the present results. As laboratory assessments were not conducted regularly, the physiological zones of each triathlete could not be accurately determined. Additionally, external factors such as training loads from swimming and running sessions, sleep duration and nutritional intake were not controlled for. Furthermore, the correlational nature of the analyses precludes any inference of causality, and the identified associations should therefore be interpreted as descriptive rather than causal. Finally, because the study followed athletes from the junior to the U23 category, part of the observed changes in the cycling power profile may be influenced by the natural physiological development associated with age. However, the average age of participants was 21 years, meaning most athletes had already completed their physiological maturation.

## 5. Conclusions

The improvements in MMP ≥ 1 min values in junior and U23 international triathletes over three consecutive seasons were associated with a higher total training volume and an increase in the time spent in moderate-intensity power bands. In contrast, increased time spent in high-intensity power bands may be related to improvements in shorter-duration MMP (10 and 30 s). The changes in MMP values ranged from 2.8% to 4.8% for durations of 1 to 40 min, and from 0.9% and 1.5% for shorter durations (10 to 30 s). These findings provide coaches and researchers with valuable insights into how cycling performance in international junior and U23 triathletes could be optimized in relation to the training volume and intensity.

## Figures and Tables

**Table 1:** Relationships between MMP values and the percentage of time spent in specific power bands during training and competition in the cycling segment among international junior and U23 triathletes over three consecutive seasons.

MMP	0–2 W·kg^−1^	2–4 W·kg^−1^	4–6 W·kg^−1^	6–8 W·kg^−1^	8–10 W·kg^−1^	10–12 W·kg^−1^	12–14 W·kg^−1^	>14 W·kg^−1^
**10 s**	−0.55 *	0.21 *	0.68 *	0.64 *	0.52 *	0.63 *	0.77 *	0.73 *
**30 s**	−0.51 *	0.19 *	0.61 *	0.66 *	0.56 *	0.64 *	0.74 *	0.65 *
**1 min**	−0.41 *	0.06	0.64 *	0.64 *	0.58 *	0.64 *	0.68 *	0.58 *
**5 min**	−0.46 *	0.17 *	0.59 *	0.57 *	0.48 *	0.54 *	0.55 *	0.48 *
**10 min**	−0.52 *	0.20 *	0.64 *	0.57 *	0.50 *	0.58 *	0.55 *	0.46 *
**20 min**	−0.50 *	0.20 *	0.61 *	0.54 *	0.49 *	0.57 *	0.56 *	0.46 *
**40 min**	−0.51 *	0.27 *	0.53 *	0.42 *	0.37 *	0.46 *	0.49 *	0.38 *
**1 h**	−0.52 *	0.23 *	0.61 *	0.51 *	0.44 *	0.52 *	0.54 *	0.48 *

* The correlation is significant at the level *p* < 0.05. A green colour scheme was used for positive correlations and a red colour scheme for negative correlations.

**Table 2:** Relationships between MMP values and the percentage of time spent in specific power bands during training and competition in the cycling segment among international junior and U23 triathletes across each season.

	MMP	0–2 W·kg^−1^	2–4 W·kg^−1^	4–6 W·kg^−1^	6–8 W·kg^−1^	8–10 W·kg^−1^	10–12 W·kg^−1^	12–14 W·kg^−1^	>14 W·kg^−1^
**1st Season**	**10 s**	−0.62 *	0.27	0.75	0.89 *	0.80 *	0.82 *	0.84 *	0.84 *
	**30 s**	−0.57 *	0.22	0.72 *	0.80 *	0.80 *	0.83 *	0.77 *	0.73 *
	**1 min**	−0.33	−0.07	0.64 *	0.73 *	0.66 *	0.63 *	0.61 *	0.59 *
	**5 min**	−0.63 *	0.21	0.86 *	0.92 *	0.82 *	0.84 *	0.83 *	0.77 *
	**10 min**	−0.69 *	0.37	0.77 *	0.81 *	0.72 *	0.78 *	0.85 *	0.74 *
	**20 min**	−0.72 *	0.44	0.78 *	0.75 *	0.61 *	0.68 *	0.74 *	0.67 *
	**40 min**	−0.82 *	0.58 *	0.79 *	0.75 *	0.69 *	0.76 *	0.72 *	0.63 *
	**1 h**	−0.79 *	0.57 *	0.75 *	0.69 *	0.59 *	0.66 *	0.66 *	0.66 *
**2nd Season**	**10 s**	−0.61 *	0.35	0.67 *	0.65 *	0.68 *	0.80 *	0.92 *	0.84 *
	**30 s**	−0.63 *	0.35	0.71 *	0.70 *	0.65 *	0.75 *	0.91 *	0.84 *
	**1 min**	−0.45	0.07	0.94 *	0.79 *	0.69 *	0.66 *	0.77 *	0.78 *
	**5 min**	−0.32	0.13	0.47	0.47	0.51	0.58 *	0.43	0.34
	**10 min**	−0.56 *	0.28	0.71 *	0.65 *	0.63 *	0.67 *	0.62 *	0.52
	**20 min**	−0.50	0.20	0.71 *	0.68 *	0.72 *	0.81 *	0.81 *	0.69 *
	**40 min**	−0.56 *	0.32	0.62 *	0.56 *	0.57 *	0.71 *	0.76 *	0.61 *
	**1 h**	−0.40	0.06	0.78 *	0.81 *	0.84 *	0.89 *	0.86 *	0.76 *
**3rd Season**	**10 s**	−0.54	0.13	0.76 *	0.72 *	0.51	0.63 *	0.85 *	0.88 *
	**30 s**	−0.65 *	0.24	0.80 *	0.73 *	0.48	0.59 *	0.83 *	0.77 *
	**1 min**	−0.50	0.06	0.84 *	0.64 *	0.50	0.68 *	0.81 *	0.73 *
	**5 min**	−0.28	0.07	0.39	0.41	0.37	0.43	0.36	0.33
	**10 min**	−0.29	0.07	0.40	0.37	0.35	0.44	0.33	0.35
	**20 min**	−0.31	0.03	0.48	0.51	0.42	0.50	0.48	0.47
	**40 min**	−0.19	0.15	0.07	0.20	0.07	0.07	0.17	0.16
	**1 h**	−0.25	0.11	0.29	0.27	0.20	0.29	0.36	0.35

* The correlation is significant at the level *p* < 0.05. A green colour scheme was used for positive correlations and a red colour scheme for negative correlations.

## Data Availability

The data that support the findings of this study are openly available upon request from the corresponding author.

## Supplementary Materials

The following supporting information can be downloaded at: https://www.mdpi.com/article/10.3390/jfmk11020138/s1. Table S1: MMP (W·kg^−1^) of seven male and female international junior and U23 triathletes across three consecutive seasons.

## Abbreviations

HIT	High-Intensity Training
MIT	Moderate-Intensity Training
MMP	Mean Maximal Power
SIT	Sprint Interval Training
