# Supplementary material for: Between-Season Changes in the Cycling Power Profile in Relation to Training Volume and Moderate-to-High Intensity in International Junior and U23 Triathletes: A Longitudinal Study
Source: J Funct Morphol Kinesiol. 2026 Mar 26;11(2):138. doi: 10.3390/jfmk11020138 (PMC13108197; doi:10.3390/jfmk11020138)
Supplement: Supplementary file 1 [file jfmk-11-00138-s001.zip › jfmk-4185341-supplementary.pdf]

Table S1. MMP ($W \cdot kg^{-1}$) of seven male and female international junior and U23 triathletes across three consecutive seasons.

		10s	30s	1min	5min	10min	20min	40min	1h
**Men**	Season 1	14.99 ± 1.91	11.41 ± 1.33	8.71 ± 1.31	6.14 ± 0.40	5.49 ± 0.25	4.94 ± 0.21	4.40 ± 0.22	4.16 ± 0.22
	Season 2	15.41 ± 1.03	11.40 ± 0.98	8.89 ± 1.18	6.06 ± 0.42	5.59 ± 0.36	5.13 ± 0.20	4.43 ± 0.27	4.07 ± 0.26
	Season 3	15.08 ± 1.04	11.77 ± 0.76	9.92 ± 0.73	6.17 ± 0.20	5.62 ± 0.25	5.30 ± 0.27	4.55 ± 0.47	4.12 ± 0.38
	**Total**	**15.16 ± 1.33**	**11.53 ± 1.03**	**9.17 ± 1.07**	**6.12 ± 0.34**	**5.56 ± 0.29**	**5.12 ± 0.22**	**4.46 ± 0.32**	**4.12 ± 0.29**
**Women**	Season 1	12.20 ± 1.28	9.37 ± 1.55	7.40 ± 0.71	5.12 ± 0.38	4.50 ± 0.29	4.23 ± 0.22	3.70 ± 0.36	3.53 ± 0.21
	Season 2	12.34 ± 0.94	9.36 ± 0.67	7.33 ± 0.77	5.60 ± 0.71	4.77 ± 0.58	4.29 ± 0.27	3.81 ± 0.23	3.46 ± 0.18
	Season 3	12.75 ± 0.75	9.20 ± 0.26	7.72 ± 1.07	5.78 ± 0.78	5.35 ± 0.71	4.82 ± 0.73	4.35 ± 0.63	3.88 ± 0.59
	**Total**	**12.43 ± 0.99**	**9.31 ± 0.83**	**7.48 ± 0.85**	**5.50 ± 0.62**	**4.87 ± 0.53**	**4.45 ± 0.40**	**3.95 ± 0.41**	**3.62 ± 0.33**
